# Etiologic and Epidemiologic Pattern of Urolithiasis in North Iran;Review of 10-Year Findings

**Published:** 2014-01-01

**Authors:** Hamid Mohammadjafari, Maryam Barzin, Ebrahim Salehifar, Mahnaz Khademi Kord, Abdolrasoule Aalaee, Roghieh Mohammadjafari

**Affiliations:** 1 Department of Pediatrics, Cellular and Molecular Research Center, Faculty of Medicine; 2 Department Of Radiology, Boalisina Hospital; 3 Department of Clinical Pharmacy, Faculty of Pharmacy; 4 Mazandaran University of Medical Sciences, Sari; 5 Faculty of Nursing, Ramsar, Iran

**Keywords:** Nephrolithiasis, Kidney Stone, Hypercalciuria, Hyperoxaluria, Cystinuria, Hypocitraturia

## Abstract

***Objective:*** To determine epidemiologic and metabolic characteristics of renal stone in the northern Iran.

***Methods:*** We prospectively analyzed demographic, clinical and metabolic findings in children less than 16 years old with renal stone revealed by ultrasonography from September 2003 to May 2012. Evaluations included serum and urine measurement of main elements predisposing patients to stone formation.

***Findings*** ***:*** 271 children (160 males) aged 2 months to 16-years (mean 30 months) were evaluated. 91 (33.6%) had a positive family history, abdominal discomfort (18.8%), UTI (11.8%) and hematuria (11.4%) were main presenting features. 45 children were diagnosed accidentally without any specific compliant. Nearly all (99%) stones lay in kidney., 35.1% had metabolic, 10% infective and 4.1% obstructive trends, 110 children had no definable etiology. Hypercalciuria (25.5%) hyperoxaluria (18.4%) and hypocitraturia (18.1%) were more frequent than uricosuria (8.5%) and cystinuria (3.1%)

***Conclusion:*** Metabolic derangement plays significant role in stone formation in our area. Patients should be carefully evaluated considering this point of view.

## Introduction

Urolithiasis is a well-known and important disorder both for severe complications and diverse etiologic conditions^[^^1^^]^. Many clinicians prefer to investigate children with stone for predisposing metabolic and anatomic factors although there are some controversies about this idea^[^^2^^-^^6^^]^. Urinary tract stone was presumed to be a rare disorder in pediatric age. It consists of only 7% of all nephrolithiasis cases seen throughout life and 1-2% of children experience the disorder

until 16^th^ year of age^[^^7^^,^^8^^]^. 

 The incidence of urolithiasis in a given population is dependent on the geographic area, racial distribution and socio economic status of the community^[^^1^^]^. Recently several reports from different countries were published that suggested an increased incidence of pediatric urolithiasis^[^^1^^,^^9^^,^^10^^]^.

 There are some important differences between developed and developing countries according to epidemiologic, etiologic, and metabolic aspects of urolithiasis. 

**Table1:** Normal laboratory values of urinary solute measurements^[^^1^^]^

**Parameter**	**Age**	**Random (mg/mg)**	**24 hr**
**Calcium**	0-6 mo	<0.8	<4mg/kg/day
	7-12mo	<0.6	
	>1yr	<0.21	
**Oxalate**	<1yr	0.15-0.26	<45mg/1.73m2/24hr
	1-5yr	0.11-0.12	
	5-12yr	0.006-0.15	
	>12yr	0.002-0.083	
**Uric acid**		<0.53mg/dl GFR	<815mg/1.73m2/24hr
**Citrate**		>180mg/gr cratinine	>140mg/1.73m2/24hr
**Cystine**		<75mg/gr creatinine	<60mg/1.73m2/24hr

 We prospectively analyzed data obtained from patients referred to our center since ten years ago to evaluate etiologic and epidemiologic pattern of urolithiasis observed in our region.

## Subjects and Methods

We prospectively observed patients referred to pediatric nephrology clinic of Boalisina hospital, a university –affiliated tertiary care center in northern Iran, from September 2003 to May 2012. Children younger than 16 years with renal stone measuring at least one equal to or greater than 2 mm were enrolled in the study. Patients with nephrocalcinosis and those with insufficient time of follow up were excluded from study. Demographic and other information including sex, age, past medical history, family history of stone disease and presenting features were obtained from interview, history taking and physical examinations. Stone diagnosis was established sonographically with 5 and 7.5 MHZ probe by two independent radiologists that were blinded to results reported by the other. Only children in whom both specialists reported nephrolithiasis were included.

 Stone size, location (e.g., kidney, ureter, and bladder) and position (e.g., upper, middle or lower third of kidney) of stones was obtained from sonographic tracing. We examined patients for metabolic factors that could lead to stone formation. Blood sample for CBC, BUN, Cr, Ca, P, Alp, Uric acid and ABG, and urine sample for Cr, Ca, oxalate, citrate, and uric acid were sent to our referral laboratory.

 Urine analysis and culture and urine qualitative test for presence of cysteine were performed in all patients. Urine as collected as a morning random specimen in younger children who couldn't cooperate for 24 hr collection. In older patients 24hr urine was collected. 

 Reference values for normal urine in our laboratory are shown in Table 1. Outcome of patients was one of spontaneous resolution or stone passage, Extracorporeal Shock Wave Lithotripsy (ESWL) or surgery. We classified stone outcome as: 1) complete cure 2) more than 50% reduction in stone size and 3) Less than 50% reduction in stone size.

**Table 2:** Initial presenting features of patients

**Presentation**	**n**	**% **
**Abdominal pain and discomfort**	51	18.8
**Urinary tract infection**	32	11.8
**Hematuria (gross/microscopic)**	31(18/13)	11.4
**Antenatal hydronephrosis**	25	9.2
**Voiding dysfunction and complaints**	23	8.5
**Diaper reddish staining**	19	7
**Vomiting**	11	4.1
**Accidental (asymptomatic)**	45	16.6
**Others**	34	12.5

**Fig. 1:**
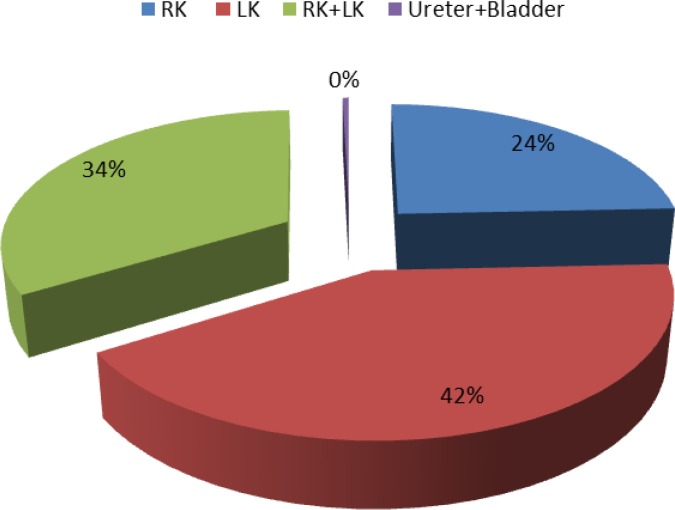
Location of stones

The beginning phase of this study was an undergraduate thesis supported by a grant from Mazandaran University of Medical sciences. Ethics committee of the said university has approved the study.

 All data was analyzed by SPSS 12 software. Statistical analysis was performed using Student t-test and Fisher’s exact test for quantitative and chi-square test for qualitative data and non-parametric tests. *P*<0.05 was considered as significant.

## Findings

We studied 271 patients 2 mo to 16 years (mean 30 months) of age. One hundred and eleven were females, and the male to female ratio was 1.44. Patients were followed at least 6 (in average 22.3) months. Abdominal pain, infection and hematuria were the leading presenting features but many patients were diagnosed incidentally (Table 2). 91 (33.6%) patients had a positive family history of urolithiasis, most commonly (9.6%) giving a history of stone in father. The stone size ranged from 2 to 17 mm with average size of 4.8±2.7 mm. Most of stones (268, 99%) lay in kidneys (Fig 1).

 Etiologically 110 (40.6%) of children had no predisposing factor. The rate of metabolic, infective and obstructive predisposing factors was 35.1%, 10% and 4.1% respectively. Twenty eight (10.3%) patients had more than one predisposing factor. Ureteropelvic junction obstruction (UPJO) in the setting of prenatal hydronephrosis was observed in 12 (4%), and was the most common obstructive disorder.

Tables 3 and 4 present the laboratory data. Hypercalciuria was the most common metabolic finding diagnosed in 25.5% of cases.

 Most of the patients received pharmacological treatment including potassium citrate (160) and thiazide diuretics (80). ESWL and surgery were done in 31 and 10 patients, respectively. Three distinct outcomes including cure or passage of stone, more than 50% reduction and less than 50% reduction in stone size, were considered at next visits done at least six months apart from the primary diagnosis, the most frequent feature being passage of stone followed by less than 50% and more than 50% reduction in stone size (Fig 2). There was no significant difference between the two sexes in size and location of stones and presence of metabolic derangement (*P*-value 0.9, 0.17 and 0.28, respectively). 

**Table 3:** Results of blood laboratory tests

**Parameter**	**Range**	**Mean ** **(** **SD)**
**Hemoglobin**	7-15.2	11.02 (1.4)
**Blood Urea**	4-52	20.5 (6.3)
**Creatinin**	0.1-1	0.59 (.11)
**Sodium (Na)**	128-148	138.4 (3.1)
**Potassium (K)**	3.3-5.96	4.39 (0.55)
**Calcium**	8.1-11.4	9.7 (0.50)
**Phosphor (P)**	3.4-7.9	5.4 (0.89)
**Alkaline Phosphatase**	126-1750	553 (230)
**Uric acid**	1.7-6.8	3.6 (0.9)
**PH**	7.23-7.59	7.39 (0.06)

SD: Standard Deviation

**Table 4:** Laboratory abnormalities observed in patients

**Abnormality**	**No of patients**	**% of patients**
**Anemia (Hgb<10)**	46	17
**Bun > 40mg/dl**	2	0.7
**Hyponatremia(Na<130mEq/L)**	2	0.7
**Hyperkalemia(K>5.5mEq/L)**	6	2.2
**Hypokalemia(K<3.5mEq/L)**	3	1.1
**Hypocalcemia(Ca<8.5 mg/dl)**	5	1.8
**Hyperuricemia(Ua>6mg/dl)**	4	1.5
**Hypocarbia (HCO**_3_ **<20meq/l)**	39	14.4
**Acidemia (pH<7.35)**	47	17.3
**Hyperphosphatemia(P>6.5mg/dl)**	22	8.1
**Hypercalciuria**	67	25.5
**Hyperoxaluria**	35	18.4
**Hypocitraturia**	37	18.1
**Uricosuria**	5 (from 53 cases)	8.5
**Cystinuria**	7	3.1

 There was no difference between patients with and without family history of metabolic abnormalities (*P*=0.61).

## Discussion

 Nephrolithiasis is an important disorder and had diverse epidemiological and etiological aspects around the world. Our region lies in geographic stone belt of Middle East but nutritional custom and lifestyle of the people resemble those of developed countries. 

 We studied near 300 children aged in average 27.5 months with male to female ratio of 1.44. This gentle male predominance was observed in most studies^[^^5^^,^^8^^,^^10^^-^^14^^]^ but recent data from Turkey and adjacent countries report higher (>2.5) male to female ratios^[^^15^^-^^19^^]^.

 Ece et al found a female predominance (M/F=29/52) that may be due to a higher frequency of stone formation associated with infection in their study population, because girls had a more frequent UTI than boys^[^^20^^]^. Edvardsson et al in Iceland showed on 26 children with urolithiasis a female to male ratio of 15/11. The female preponderance in their study was explained by a more common occurrence of hypercalciuria and hyperoxaluria in girls but no gender difference was noted in the number of patients with other anomalies^[^^21^^]^. Similar findings were reported by Mortazavi and coworker^[^^8^^]^ (2007) and Bush et al^[^^13^^]^ (2009) with no significant reason. 

 We found no significant differences between two sexes in terms of size, location and presence of metabolic disorder. There were only 3 lower tract stones in our study population and therefore relation between sex and location statistically may not be true.

 Aggour showed that male patients had more lower urinary tract stones, 22% of their l00 children had lower tract calculi^[^^19^^]^.

 In study of Bastug, while hypercalciuria and hyperoxaluria were more common in girls and hyperuricosuria and cystinuria were common in boys, the difference was not significant^[^^12^^]^.

 There are studies that found no significant difference between two sexes regarding the bladder stone^[^^11^^]^.

 Most published data revealed that abdominal pain and gross or microscopic hematuria are common presenting features of nephrolithiasis in children and adolescents^[^^18^^,^^21^^-^^25^^]^. In our study 17.4% of patients had abdominal pain and 11.4% hematuria.

 Safaei and coworkers showed that 20 children of their 84 study population presented with symptoms of UTI and the urinary calculus was diagnosed during imaging studies for UTI follow up^[^^14^^]^. 

 In our study, 53 children presented accidentally without any specific signs or symptoms related to stone disorder or presented with nonspecific signs or symptoms. In another Iranian study in 184 children with nephrolithiasis, the most frequent symptoms at presentation were symptoms of UTI in 46 (25%) and nonspecific symptoms such as irritability and restlessness in 44(24%) patients^[^^8^^]^.

 Coward studied 121 children and observed that 17% had no symptoms or signs^[^^26^^]^. Accidental stone findings were also reported by Safaei (8.3%) Gurgoze (8.9%) and Bastug (13%)^[^^10^^,^^14^^,^^12^^]^. 

 10% of our cases were diagnosed following up antenatal hydronephrosis, a presentation that was not mentioned in any other study. Our center acts as a referral hospital in Northern Iran for diagnosis and follow-up of prenatal hydronephrosis. This finding mandates close monitoring of these infants for long term complications.

 Another unusual finding in our patients was reddish-brown diaper stainining, a discoloration caused by urate crystals that casn be observed in normal babies too. The high frequency of this phenomenon suggests to perform renal imaging in all of them. Another study with different methods is needed to answer this question.

 Most of renal stones lie in the upper urinary tract, kidney. The frequency of ureter and bladder stone was reported 15 to 30% in different papers^[^^7^^,^^11^^,^^19^^-^^21^^,^^26^^]^. In our study only 3 patients had lower tract calculi, a finding that is unique. This is probably due to lower usage of CT scan in our center. In relatively old study of Tekin et al, stones were localized in the upper urinary tract in all 78 patients but 1^[^^23^^]^. The frequency of lower tract stones was reported by Gurgoze et al as 1.8% and by Safaei and Yusuf Sari as 6%^[^^10^^,^^14^^]^. If we consider bladder stone as a marker of endemic and developing regions, these three reports from Iran and Turkey are contradictory^[^^18^^]^. 

 Metabolic urine derangement was common in our samples similar to that found in many other studies^[^^14^^-^^16^^,^^22^^,^^24^^]^. 

 Cystinuria and uricosuria are not common findings but hypercalciuria, hyperoxaluria and hypocitraturia are common and important metabolic predisposing factors in different countries.

 Hypocitraturia was the most common urinary abnormalitiy in recent Turkish studies^[^^10^^,^^22^^]^ and also studies performed by Rizvi et al in Pakistan^[^^18^^]^ and Kalorin et al in Albany^[^^25^^]^. On the other hand, other Turkish studies^[^^19^^,^^12^^]^ and recent Iranian studies^[^^8^^,^^14^^]^ reported hypercalciuria as most frequent urinary findings.

 Aggour et al found hypercalciuria in 37.1% of patients^[^^19^^]^ and Coward^[^^26^^]^ reported this abnormality in 57% of children with stone. Similarly hypercalciuria and hyperoxaluria were two common urinary findings in our patients and frequency of uricosuria was very low.

 Only 40% of our cases had normal findings on laboratory evaluation, therefore it is important to investigate every child with urinary stone disorder for underlying metabolic derangements.

## Conclusion

Nephrolithiasis is an important problem in our area. Stone distribution resembles that observed in developed countries and similarly metabolic factors are common. This mandates closed and complete evaluation of children presented with urinary tract stone. Unexpectedly large percentage of stone presentations are subtle clinical states such as diaper staining and other nonspecific complaints. These issues need to be carefully considered and followed up for more precise diagnosis and treatment.
